# Structural colour of unary and binary colloidal crystals probed by scanning transmission X-ray microscopy and optical microscopy

**DOI:** 10.1038/s41598-017-12831-4

**Published:** 2017-09-29

**Authors:** Hyun Woo Nho, Tae Hyun Yoon

**Affiliations:** 1 Department of Chemistry, College of Natural Sciences, Research Institute for Natural Sciences, Hanyang University, Seoul, 04762 Republic of Korea; 2 Present Address: LG Chem R&D Campus Daejeon, Daejeon, 34122 Republic of Korea

## Abstract

Colloidal crystals composed of micro- or nano- colloids have been investigated in various fields such as photonics due to their unique optical properties. Binary colloidal crystals have an outstanding potential for fine-tuning material properties by changing the components, concentration, or size of colloids. Because of their tunable optical, electrical, magnetic, and mechanical properties, those materials attracted great attention. However, it has been hard to elucidate internal structures without fluorescent labelling or cross-sectioning. Here, we demonstrate the structural analysis of not only unary but also binary colloidal crystals using scanning transmission x-ray microscopy and compare the results with colloidal structures and optical properties observed by optical microscopy. Based on the comparison of images obtained by these two methods, the domains of colloidal crystals consisting of different structures and colours were directly identified without any additional sample preparation. Therefore, it was possible to investigate the structural colours of local domains of unary and binary colloidal crystals such as the face centred cubic (FCC) structure with different orientations, that is FCC (111) and FCC (001), and hexagonal close-packed structure, HCP (0001).

## Introduction

Two- or three- dimensional colloidal crystals have been studied extensively because of their unique optical properties and potential for various industrial applications such as optical devices (waveguides, flexible polymer colloidal crystal lasers)^1–6^ and sensors (pH, volatile organic solvent, humidity)^7–13^. Due to the interference phenomena, light waves with similar wavelength to the period of colloidal structures often show selectively inhibited light propagation and are known as photonic band gap or photonic stop band. Recently, an omnidirectional photonic band gap, which was not observed in typical unary colloidal crystals composed of PS or SiO_2_ particles due to their low dielectric contrast, was reported in binary colloidal crystals or unary colloidal crystals with inverse opal structure and has attracted significant attention. Binary colloidal crystals consist of particles with two different sizes and compositions, while inverse opal structures comprise particles and an “air sphere”^14^. Since unary colloidal crystals can be used as template for binary colloidal crystals or inverse opals, the structural characterization of unary colloidal crystals is important. Although the most common structure of unary colloidal crystals is the face centred cubic (FCC) structure, plane stacking faults or multiple domains are frequently observed because of the small difference of free energy (~10^−3^ k_B_ per sphere) between FCC and hexagonal close-packed structures (HCP)^15^.

X-ray microscopy (XRM) can be used complementary to conventional imaging techniques for photonic crystals such as electron microscopy (EM) and optical microscopy (OM). The latter two methods have been used as general techniques for the investigation of colloidal crystals. These microscopic techniques have advantages and disadvantages with respect to the characterization of colloidal crystals. For example, colloidal crystals can be observed with the highest spatial resolution using EM; on the other hand, limited (thinner than 0.5–1 µm) sample thickness^16^ or surface observation is inevitable for transmission electron microscopy (TEM) or scanning transmission electron microscopy (SEM), respectively. The spatial resolution of OM is not high enough to investigate the structure of colloidal crystals; therefore, advanced fluorescent optical microscopy, such as laser scanning confocal microscopy or stimulated emission depletion microscopy, have been used to characterize structures of colloidal crystals with better resolution^17^. However, for these fluorescence microscopic techniques, colloidal particles have to be labelled with fluorescent dyes and immersed in solution for refractive index matching. Therefore, complementary microscopic techniques with of high spatial resolution capabilities, good penetration depth (>1 µm), and label-free imaging capability are necessary. Synchrotron-based X-ray microscopy (XRM, i.e., scanning transmission X-ray microscopy, STXM) fulfils those requirements and has been used to investigate colloidal crystal structures^18–23^.

The structural colour of colloidal crystals is one of the unique characteristics of colloidal crystals useful for various optical applications. Generally, spectroscopy (reflectance or transmittance spectra) has been used to characterize the optical properties of colloidal crystals. However, it is difficult to grow single-domain colloidal crystals experimentally, which is mainly due to the small differences of the free energy between crystal structures^15^. Therefore, spectra represent averaged characteristics of multiple domains of colloidal crystals because the light source with a beam diameter of a few millimetres covers multiple domains. It is not possible to differentiate domains of different structures using spectroscopy. Thus, lots of studies considering the structure and colours have been restricted to theory due to the limitations of observation tools.

In this study, unary and binary colloidal crystals were prepared and characterized using both scanning transmission X-ray microscopy (STXM) and optical microscopy. This is the first study reporting the observation of structures of binary colloidal crystals using STXM and directly identifying correlations between their colours and real structures. The colloidal crystals were fabricated with a modified spin coating method using a mixture of small and large colloidal particle-suspended solution. The internal structures of colloidal crystals were investigated using STXM and the structural colours of different domains were observed with optical microscopy. We can easily and directly match colloidal structures and colours using these two techniques.

## Experimental Section

### Fabrication of colloidal crystals for STXM observation

**Figure 1:**
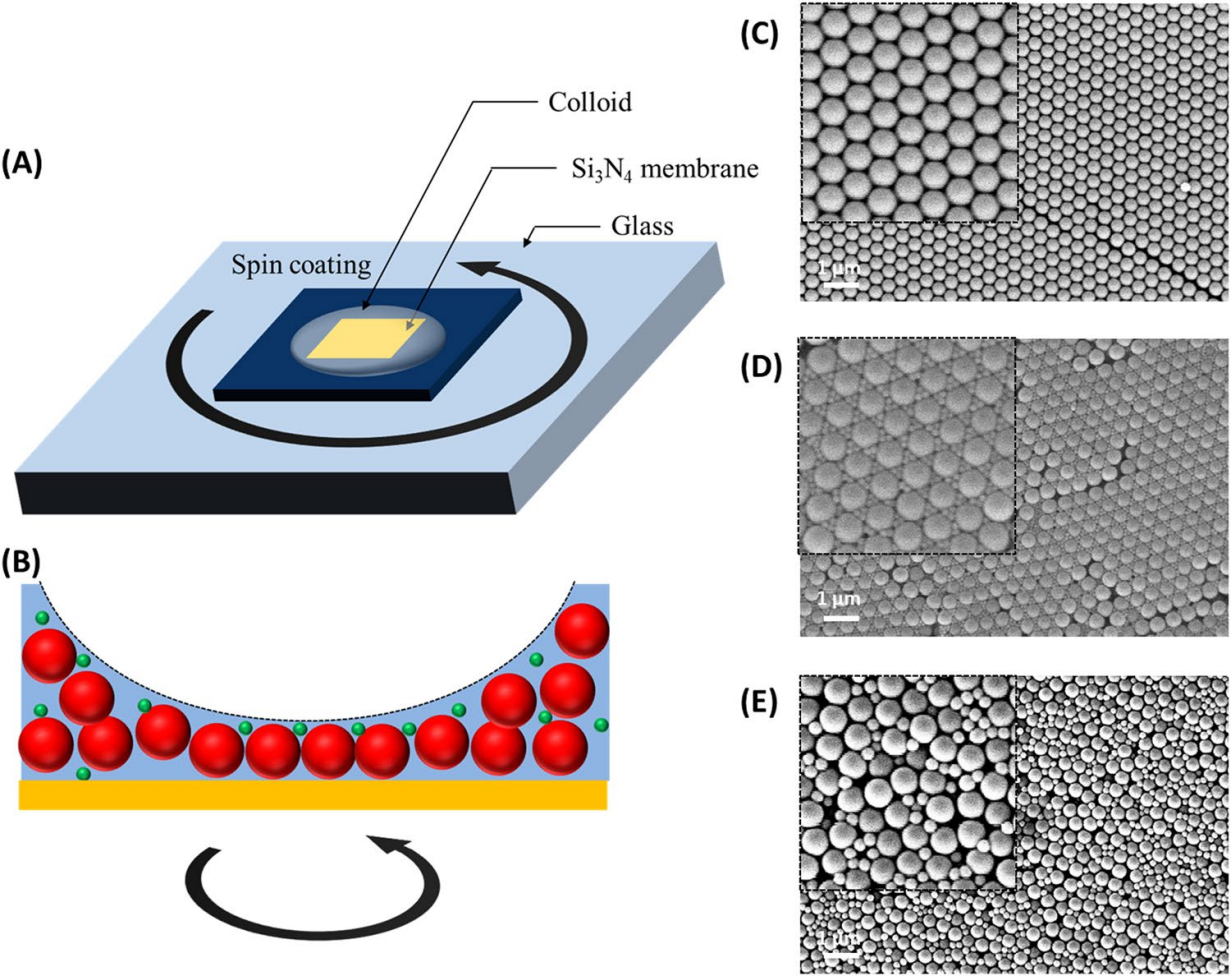
(**A**) Schematic illustration of the spin coating method for the fabrication of binary colloidal crystals and (**B**) cross-sectional view of the fabricated binary colloidal crystal on the Si_3_N_4_ membrane substrate. SEM images of (**C**) unary colloidal crystals consisting of polystyrene (PS) with 500 nm colloids, (**D**) binary colloidal crystals with 500 nm PS and 100 nm SiO_2_, and (**E**) amorphous colloidal mixture with 500 nm PS and 200 nm SiO_2_.

As illustrated in Fig. 1A, Colloidal crystals were fabricated on the Si_3_N_4_ membrane (100 nm thickness) substrate (NX5150 C, Norcada Co., Edmonton, AB, Canada) for STXM analysis. We purchased 500 nm polystyrene (PS; ~25 mg/mL; Polysciences, Warington, PA, USA), which was spin-coated on the Si_3_N_4_ membrane substrate. Before spin coating of the PS solution, the Si_3_N_4_ substrate was treated with O_2_ plasma (100 W, 0.2–1 mbar, 45 s; CUTE, Femto Science, Gyeonggi-Do, Republic of Korea) to clean the surface. The frame of the Si_3_N_4_ substrate was attached to the glass substrate by using kapton tape for spin coating because the Si_3_N_4_ substrate was too small (5 × 5 mm frame and 1.5 × 1.5 mm membrane) to be mounted on the chuck of the spin coater. Subsequently, 5 μL of the PS colloidal solution was put on the Si_3_N_4_ substrate, spin-coated at 2000 rpm for 15 s, and then ramped up at 3500 rpm for 600 s.

Binary colloidal crystals were also fabricated using the spin coating method: 100 μL of 25 mg/ml, 500 nm PS solution was mixed with 100 μL of 5 mg/ml, 100 nm SiO_2_ solution (PSI-0.1, Kisker Biotech GmbH & Co. KG, Steinfurt, Germany). The other procedures (O_2_ plasma treatment and spin coating) were the same as the fabrication of unary colloidal crystals.

### Observation of colloidal crystal using STXM and optical microscopy (OM)

**Figure 2:**
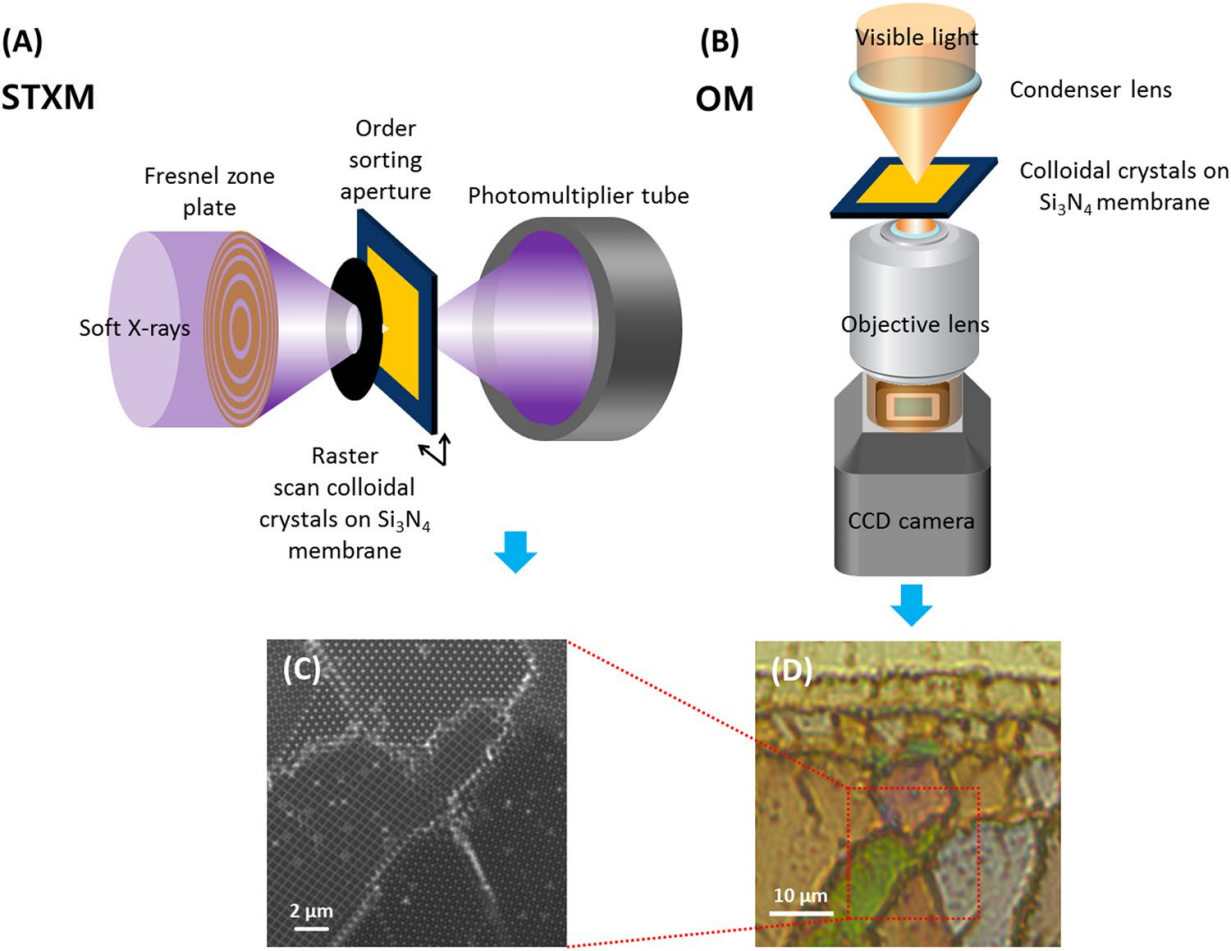
Schematic illustration of (**A**) scanning transmission X-ray microscopy (STXM) and (**B**) optical microscopy (OM). Colloidal crystal images measured by (**C**) STXM (transmission mode measured at 500 eV) and (**D**) OM (transmission mode).

The colloidal crystals were analysed with an optical microscope (DM6000M, Leica, Germany) equipped with a colour CCD (DFC 550, Leica, Germany) and synchrotron-based STXM. The schematic illustration of the OM and STXM setup is shown in Fig. 2. OM images were acquired with 50x or 10x objective lenses with numerical aperture (NA) of 0.8 and 0.3, respectively. (see ESI-Figure 1) STXM image was acquired at the 10 A beamline of the Pohang light source (PLS, Pohang Accelerator Laboratory, Republic of Korea) and 10ID-1 (SM) beamline of the Canadian light source (CLS, University of Saskatchewan, Canada). The storage ring current of 300 mA at the PLS was operated in top-up mode (SR energy of 3.0 GeV). The storage ring current was 250 mA in decay mode (SR Energy of 2.9 GeV) at the CLS. The synchrotron-based monochromatic soft X-ray was focused to ~40 nm using the Fresnel zone plate. The first order of a diffractive focused X-ray was selected using the order-sorting aperture (OSA, pinhole of 100 µm). The sample was mounted on the interferometrically controlled piezo stage and raster scanned with a 20-nm step size. The intensity of transmitted X-ray was measured using a scintillator photomultiplier tube (PMT). The unary colloidal crystals consisting of 500 nm PS were observed at 500 eV. The binary colloidal crystals made of 100 nm SiO_2_ and 500 nm PS were investigated at 280 eV for SiO_2_ and at 500 eV for PS. The aXis2000, a software for x-ray image and spectra analysis, was used to calculate the optical density (OD) of the binary colloidal crystals consisting of PS and SiO_2_ (aXis2000, http://unicorn.mcmaster.ca/aXis2000.html, Hitchcock A. P., 2012), while the Trak EM2 of Fiji (open source image processing platform)^24^ was used to align and overlay pseudocolours on the two images obtained at different photon energies.

## Results and Discussion

### Fabrication of unary and binary colloidal crystals

The 3D colloidal crystals were fabricated by spin coating. In other studies, layer-by-layer methods were introduced for the fabrication of binary colloidal crystals^25,26^. Lager particles were firstly spin-coated on the substrate and then smaller particles were spin-coated on the interstices of the lager particles. Based on this approach, monolayer or bilayer colloidal crystals could be easily fabricated; however, the fabrication of multilayered or 3D colloidal crystals was difficult, since the predeposited particles were easily peeled off. In this study, co-crystallization method using spin coating technique was employed for the single step fabrication of multilayered colloidal crystals. Because there was the step height (~100 μm) between the substrate and kapton tapes for the fixation of the substrate, the colloidal solution was kept thick enough for the formation of multiple layers (Fig. 1B). Using this method, not only unary colloidal crystals (Fig. 1C) but also multiple layers of binary colloidal crystals were fabricated (Fig. 1D and E) in the vicinity of kapton tapes. However, as described in other studies, the size ratio [γ_S/L_, diameter ratio between small (S) and large (L) particles] of binary colloidal crystals is theoretically limited below 0.225^27^. This was experimentally confirmed, as shown in Fig. 1C–E. When γ_S/L_ was 0.2, three small SiO_2_ particles were closely packed in the interstices of large PS particles (Fig. 1D). However, when γ_S/L_ was increased to 0.4, the structure changed to an irregular amorphous structure (Fig. 1E). Based on these results, we confirmed the fast, simple, and easy fabrication of multilayered binary colloidal crystals assembled by spin coating of the suspension of large and small colloidal particles.

### Characterization of colloidal crystals using STXM and optical microscopy (OM)

The domain of colloidal crystals can be distinguished based on the different colours in OM transmission images (Fig. 2D). These results are not limited to our observations; in other studies, similar images were found^28–30^. Some domains are brownish-green, others show pale-purple or yellow–ochre colours. In addition, identical regions were investigated with STXM to observe colloidal crystal structures with high enough spatial resolution for the characterization of individual particles or interstices (Fig. 2C). Because the OM image was measured in transmission mode, the colours representative of colloidal crystal domains match the complementary colours of the stop band or photonic band gap. In other words, red light cannot penetrate green domain and navy or blue colours cannot infiltrate the yellowish domain and vice versa. In spectroscopic studies, the transmission or reflectance peak position is often calculated based on the modified Bragg equation^31,32^.

$${\rm{m}}{\lambda }=2{\rm{d}}{({{{\rm{n}}}_{{\rm{eff}}}}^{2}\mbox{--}{\sin }^{{\rm{2}}}{\rm{\theta }})}^{1/2},$$

where m is the order of diffraction; λ is the wavelength; d is an interplanar distance that is determined by the diameter of the particle (D), that is, (2/3)^1/2^D for face-centred cubic (FCC) and hexagonal close-packed (HCP) structures; n_eff_ is the effective refractive index, 1.44 for PS based on n_eff_ = n_PS_Φ + n_air_(1 – Φ), where Φ is the filling fraction of the particles and 0.74 for FCC and HCP; and θ is the incident angle of light. Based on this equation, the diffractive colours of FCC (111) and HCP (0001) should be identical and those structures cannot be distinguished based on the colour. However, the FCC and HCP domains have different colours in the OM image. If the colloidal particles consist of 500 nm PS, the second order of the diffractive wavelength of the FCC structure is 587 nm (yellow) and the transmitted light should be bluish.

Figure 2C shows that structures consisting of different domains, such as FCC (111), FCC (100), and HCP (0001), could be identified using STXM. In addition, internal defects were directly observed in real space^18,19^. Based on those two images (Fig. 2C and D) and the observed identical regions using STXM and OM, we could directly correlate the colloidal structures with their colours. The FCC (100), FCC (111), and HCP (0001) domains show brownish-green, pale-purple, and yellow–ochre colours, respectively. As stated above, although the conditions for the fabrication of single-crystalline colloidal crystals were proposed in several reports^33,34^, it is still hard to grow single-crystal domains due to the small differences of the free energy of FCC and HCP (10^−3^ k_B_ per sphere)^15^. Various domains of colloidal crystals have different stacking sequences; therefore, structural characterization is fundamental to reveal the relation between optical properties and the crystal structure.

There have been assumption and theoretical simulation results that different colours of colloidal crystals correlate with distinct structures or orientations^28,32^. Therefore, structures of colloidal crystals, such as FCC (100) and FCC (111), can be inferred based on different colours. However, most techniques for the characterization of colloidal crystals cannot directly detect differences between FCC and HCP in the real space. Most studies focused on FCC structures of self-assembled colloidal crystals^33,35–38^. Scanning electron microscopy is commonly used to characterize colloidal structures because of the superior spatial resolution. However, only the top surface can be observed with SEM such that the sample should be cross-sectioned to observe internal structures and sequences to confirm if the structure is FCC or HCP. Therefore, FCC and HCP structures cannot be identified in SEM images without destructive sample preparation. In addition, conductive metallic coating is hardly avoidable, which can change the optical properties. Another common tool to characterize colloidal crystals is confocal scanning laser microscopy (CSLM), which compensates for the disadvantage of OM, the spatial resolution, by adopting pinholes to block the light that is out of focus^39^. However, the colloids have to be labelled with fluorescent dyes to investigate their crystalline structure using CSLM. Furthermore, colloidal crystals have to be immersed in a proper solution for refractive index matching^39,40^. Therefore, it is difficult to observe colloidal crystals under dry conditions. However, by using STXM and OM, the real structure can be identified without destructive sample preparation or fluorescent labelling. In addition, the FCC and HCP structures can be differentiated based on the different colours, that is, pale purple and yellow ochre or greenish colours.

**Figure 3:**
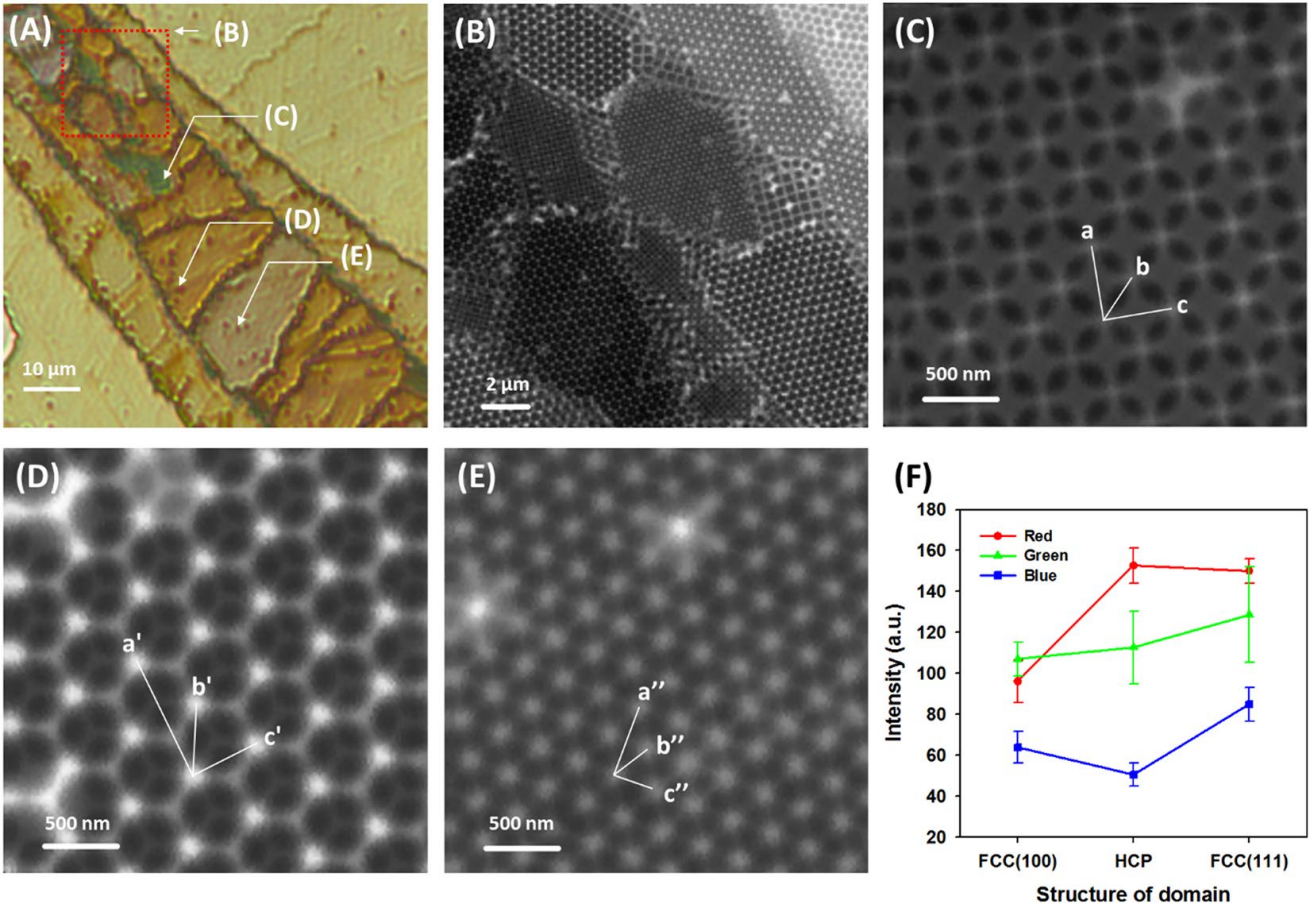
(**A**) OM image (transmission mode) of the four-layered colloidal crystal. Green, dark goldenrod, and ivory colours were observed in different domains. (**B**) STXM transmission image acquired in the region marked with a red dashed line in (**A**). (**C**) STXM image of the FCC (100) domain (green), (**D**) HCP (0001) domain (dark goldenrod), and (**F**) FCC (111) domain (ivory). (**F**) Red, green, and blue colour intensity of each domain of (**C**), (**D**), and (**E**).

To confirm the correlation between the colours observed by OM and colloidal crystal structures measured by STXM, another location of the colloidal crystals was investigated and the details of the structures were identified (Fig. 3). Synchrotron-based X-ray microscopy is a versatile tool for the observation of colloidal crystals because of the high spatial resolution, significant penetration depth, and chemical speciation capability. Although the measurement speed of STXM is lower than that of TXM, the field of view (FOV) or magnification can be varied in STXM. Therefore, it is much easier to compare the OM and STXM images of identical regions to determine correlations between the colour and colloidal structure by measuring STXM images at relatively low magnifications and then increasing the magnifications step by step. Figure 3 shows that the detailed structure of each domain was directly characterized using STXM and the spatial resolution was high enough to identify the most common structures of individual colloidal particles such as the FCC (100), FCC (111), and HCP (0001) structures (Fig. 3C–E). Based on the comparison of the colourful OM images, we could infer the colloidal structures of each domain in a large FOV. The OM image in Fig. 3A has a lower optical densities compared with Fig. 2D because of the smaller number of layers of the colloidal crystal. Similar to other studies, thick colloidal crystals (more than four layers) have a high chromaticness because the number of layers of the colloidal crystal affects the width of the photonic stop band and optical density^34,41^. Such trends are also evident in the OM images (Figs 2D and 3A). When increasing the number of layers, dark and vivid colours were observed, although the structure of the colloidal crystal was identical. The number of layers measured by STXM in Figure 2D is four. However, in Fig. 3D and E, the HCP and FCC structures showed only three layers. Therefore, the colour of the structures in Fig. 3A are dark goldenrod and ivory, respectively. The colour of the domains in Fig. 3C–E are represented as average red, green, and blue (RGB) colour intensity and their distributions are shown in Fig. 3F. Based on the RGB intensity, we obtained more quantitative colour information from the OM image. In the FCC (100) and HCP (0001) domains, the green and red colour components were relatively higher than the blue colour component. In the FCC (111) domain, the blue colour component was relatively higher than that of other two structures, although red and green colour components were still dominant. In addition, the number of layers of the colloidal crystal also represents the different RGB values (ESI-Figure 2 **)**. Each pixel of the OM image was plotted in RGB colour space using a frequency-weighted mode. The mono, double, and triple layers were clustered in different groups and structures such as FCC (100), FCC (111), and HCP (0001). The RGB measurement result for the FCC (111) domain is in partially agreement with the calculation based on the modified Bragg equation. As mentioned above, the transmitted colour of FCC (111) should be bluer than that of other structures. In addition, the RGB values of HCP (0001) and FCC (111) differ, which cannot be explained by the modified Bragg equation.

Internal defects also affect the optical properties of colloidal crystals. To verify the influence of defects on the optical properties, optical transmission, reflection, and diffraction studies were performed in the past^29,42^. Defects or stacking faults induce the broadening or splitting of transmission peaks or reflection spectra, known as photonic stop band. However, there are other causes for the broadening of the photonic stop band such as inhomogeneous thickness and multiple domains with different orientations of colloidal crystals. With increasing thickness of the colloidal crystal, the width of the stop band decreases^34,41^. In addition, different wavelengths of incident light are attenuated in different domains with a distinct orientation based on the Bragg equation. Therefore, although theoretical simulation studies of optical properties of colloidal crystals are well established, the experimental work is still in the beginning stages. It is difficult to determine which disorder (defects or different crystal orientation) of colloidal crystals contributes to the broadening or splitting of the stop band with spectroscopic studies using a beam with a diameter of a few millimetres. Moreover, local defects within the colloidal crystals cannot be characterized well. However, defects representing distinct colours (dark reddish colours) are easily recognized in OM images (Fig. 3A) and detail structures of those defects can be observed in STXM images (Fig. 3B–E). Therefore, orientations and defect effects showing similar optical properties can be manifested in multi-domain colloidal crystals using OM and STXM imaging.

In our previous study^23^, structures of colloidal crystals of 500 nm PS were analysed using full field transmission X-ray microscopy (TXM), where we distinguished fcc and hcp 3D structures with thickness of 3 or 4 layer via comparisons of their line profiles of optical densities with those simulated from model structures. Similarly, STXM can also provide us 3D colloidal crystal structures with information along the normal axis as well as lateral direction, which can be compared with the optical transmission data from OM. In STXM, each structure was analysed by measuring the void distances of each structure of colloidal crystals and comparing theoretical distances. The void distances marked as a, b, and c in the FCC (100) plane; a’, b’, and c’ in the HCP (0001) plane; and a”, b”, c” in the FCC (111) plane were measured (Fig. 3C–E) and compared with theoretical distances. The detailed values are represented in ESI-Table 1. The differences between theoretical and measured distances were less than 8% and originated from the size deviation of colloids and stacking faults^21^.

### Analysis of binary colloidal crystals

**Figure 4:**
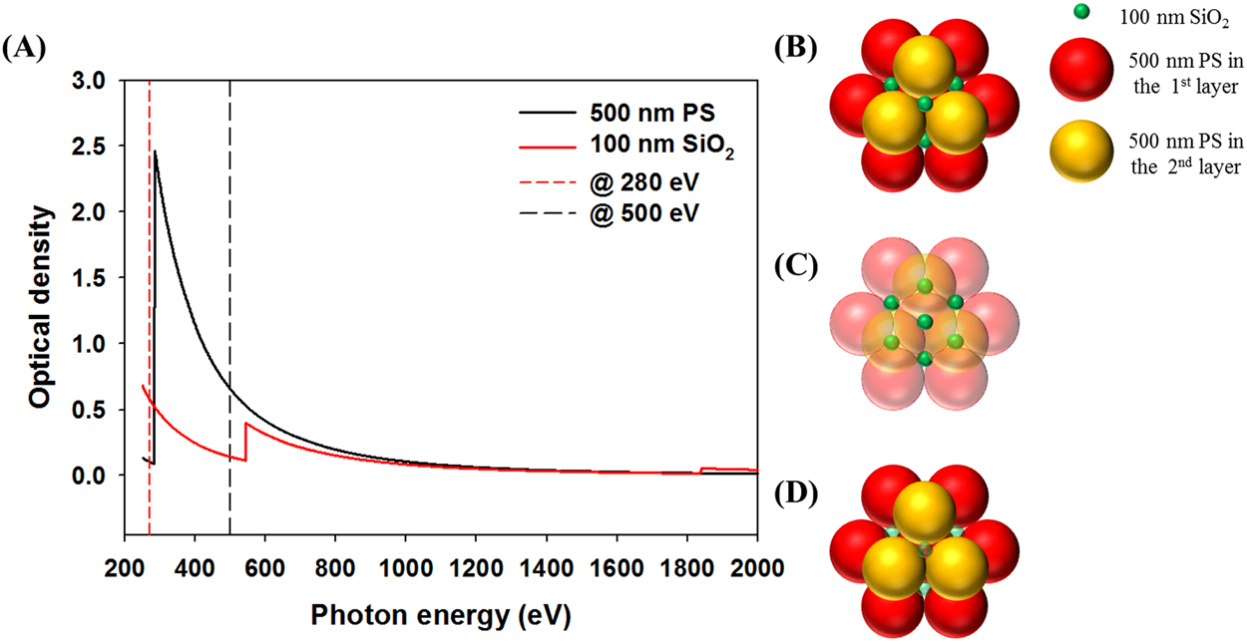
(**A**) Simulated optical density (OD) for binary colloidal crystals consisting of 500 nm PS and 100 nm SiO_2_. The black and red lines indicate the OD of the PS and SiO_2_ colloids, respectively. (**B**) Model structure of binary colloidal crystals of the double layers. Expected figurations of STXM measured (**C**) at 280 eV (pre-edge of C K-edge) and (**D**) at 500 eV (pre-edge of O K-edge).

**Figure 5:**
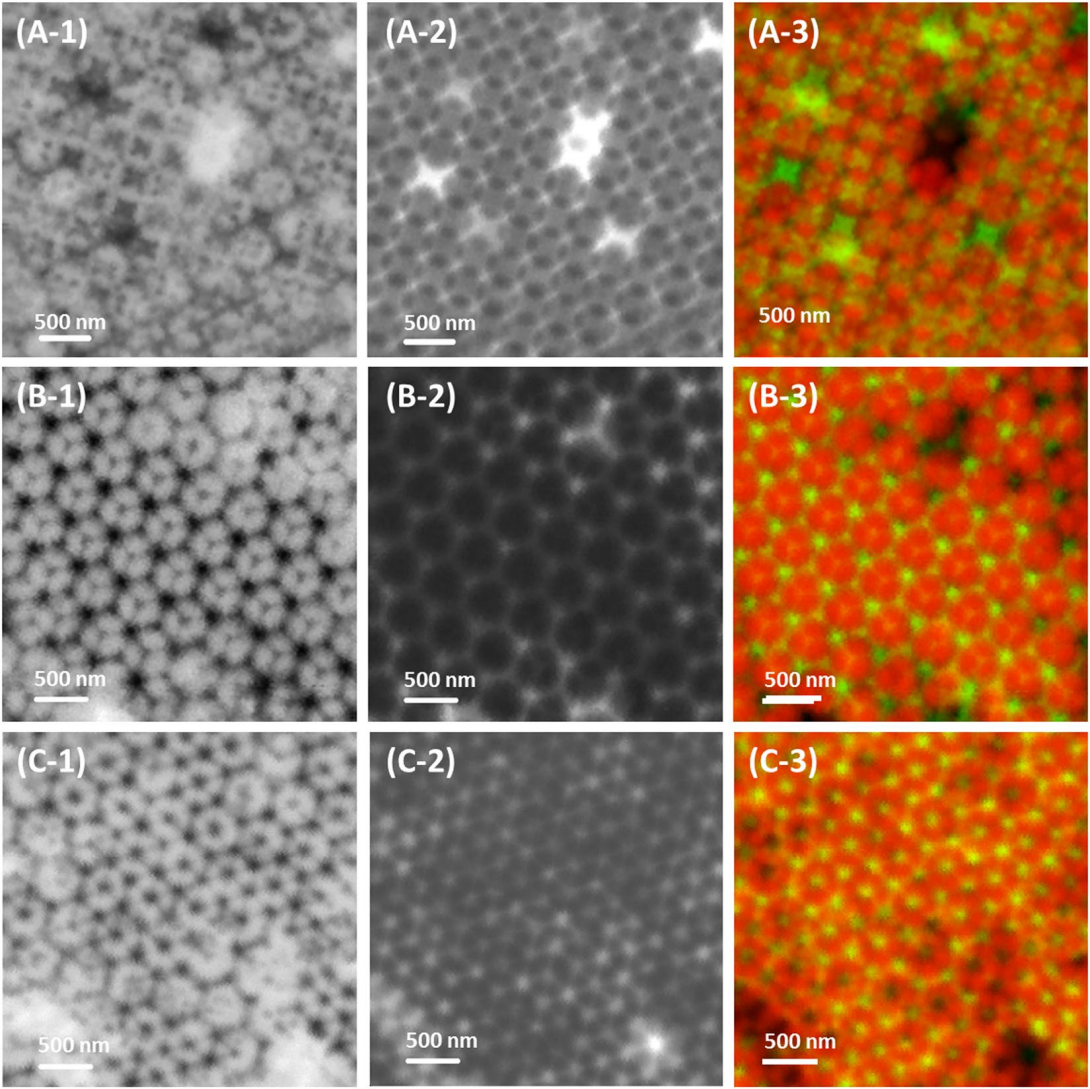
(**A**) STXM transmission images of the FCC (100) domain measured (A-1) at 280 eV for the observation of SiO_2_ and (A-2) at 500 eV for the analysis of PS colloids in binary colloidal crystals. (A-3) Pseudocolour image obtained by merging (A-1) and (A-2). (**B**) STXM transmission image of the HCP (0001) domain (B-1) observed at 280 eV for SiO_2_ and (B-2) at 500 eV for PS. (B-3) Pseudocolour image obtained by merging (B-1) and (B-2). (**C**) FCC (111) planes measured in STXM transmission mode at (C-1) 280 eV for SiO_2_ and (B-2) at 500 eV for PS. (B-3) Pseudocolour image obtained by merging (C-1) and (C-2).

The characterization of binary colloidal crystals was one of challenges of this study because of the complexity of their structures, heterogeneity of the colloids, and limitations of the characterization method. However, one of advantages of STXM is the capability of chemical speciation and elemental mapping via acquiring images at different photon energies. Therefore, chemical contrast images of two different elements were readily acquired for binary colloidal crystals consisting of different colloid components. As illustrated in Fig. 4, PS and SiO_2_ particles have a different X-ray absorption contrast at the C K-edge (285 eV, C = C 1π*), O K-edge (544 eV), and Si K-edge (1845 eV). At the pre-edge of the C K- or O K-edge, carbon or oxygen are relatively transparent, respectively. Consequently, only SiO_2_ particles can be observed at the pre-edge of the C K-edge (Fig. 4C) and only PS particles can be measured at the pre-edge of the O K-edge (Fig. 4D) with high contrast. Therefore, as shown in Fig. 5, 100 nm particles of SiO_2_ (Fig. 5A-1, B-1, and C-1) and 500 nm PS particles (Fig. 5A-2, B-2, and C-2) could be classified by observations at 280 eV and 500 eV, respectively. The STXM images for SiO_2_ and PS colloidal crystals were merged to represent both structures simultaneously [Fig. 5A-3 for FCC (100), 5B-3 for HCP (0001), and 5C-3 for FCC (111)] using pseudocolours (i.e., green for SiO_2_ and red for PS).

The larger PS particles are templates and the smaller SiO_2_ particles fill the interstices between the PS particles. Because the void types (i.e., tetrahedral or octahedral void) in colloidal crystals originate from colloidal structures of larger PS particles, the colloidal structures of SiO_2_ particles are determined based on the colloidal structure of the PS particles. Thus, the discrepancies of the three-dimensional colloidal structures of SiO_2_ are induced by FCC (111) and HCP (0001) structures of PS particles having different combinations of voids. For instance, the peak-to-peak distance between most bright positions (void blocked by single PS particle) in the FCC (111) structure is 1/√3 times the diameter of the PS and the radius ratio [maximum radius filling the void (r)/radius of PS (R)] is 0.225 in the tetrahedral void. However, in the HCP (0001) structure, the peak-to-peak distance between most bright open voids is √3 times the diameter of the PS and the radius ratio (r/R) is 0.414 in the octahedral void. Therefore, the volume of the octahedral void is approximately 6-fold larger than that of the tetrahedral void. Although the volume is different between the tetrahedral and octahedral voids (Fig. 5C-2), the bright position (combination of tetrahedral and octahedral voids, see ESI-Figure 3) was evenly distributed in FCC (111) structures and SiO_2_ particles filled those voids equally (Fig. 5C-1) because the combination of voids between the first and second layer (tetrahedral–octahedral or tetrahedral–tetrahedral voids) reduces the volume differences to 3.5-fold. However, the HCP structure contains combined open holes (eclipse-shaped octahedral voids), which are filled with a larger number of SiO_2_ particles due to the volume differences remaining approximately 6-fold. Therefore, the SiO_2_ particles in octahedral voids (Fig. 5B-1 and B-3) were more remarkable with higher contrast than those in the tetrahedral voids. This indicates why the FCC (111) and HCP (0001) structures show different colours. Although the total number of voids is identical (two tetrahedral and one octahedral voids per colloid)^15^, combinations or sequences are different in the FCC or HCP structures. In the HCP structure, octahedral–octahedral voids have a localized larger void volume (~6-fold) than tetrahedral–tetrahedral voids in the FCC structure. Moreover, in the HCP structure, light is able to pass without change of the refractive index from octahedral–octahedral voids because these voids are connected. Therefore, the effective refractive index, n_eff_, of the modified Bragg equation should be considered for the local void types in three-dimensional colloidal crystals. The refractive index n_eff_ should be smaller in the HCP structure than in the FCC structure because the local void volume of eclipse-shaped octahedral voids is larger and reflected λ becomes smaller than in FCC. Hence, the transmitted light is shifted to red. Otherwise, n_eff_ is higher in the FCC structure than in the HCP structure; therefore, the transmitted colour is bluish.

**Figure 6:**
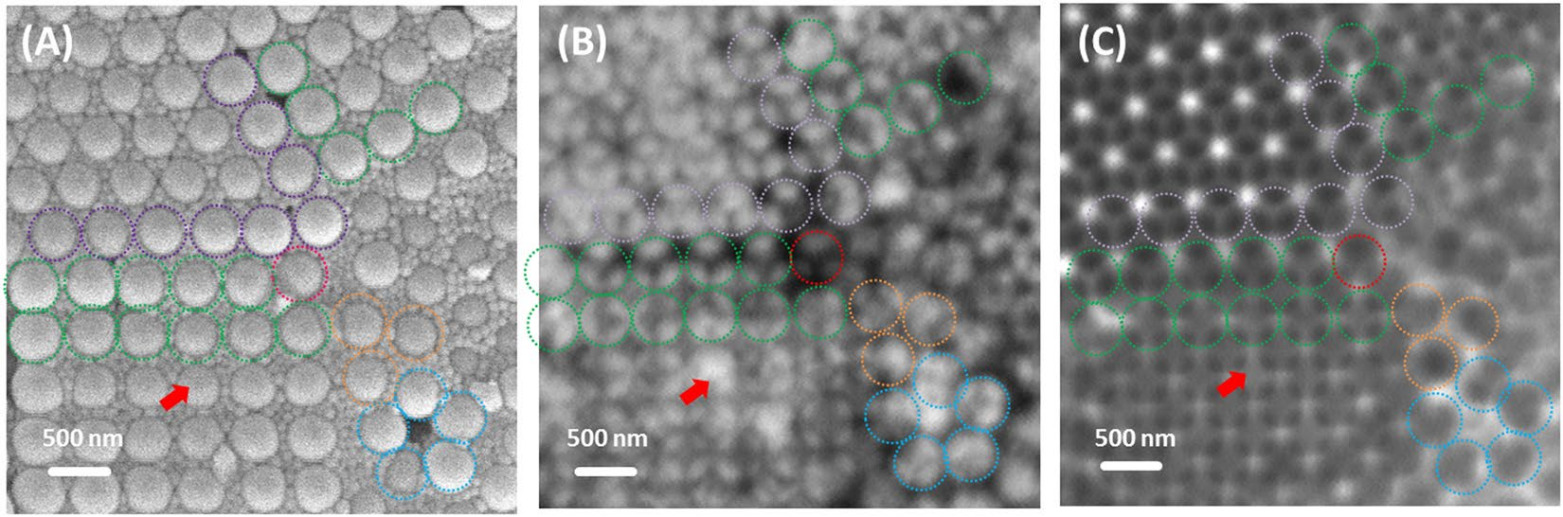
(**A**) SEM image of binary colloidal crystals consisting of 100 nm SiO_2_ and 500 nm PS. STXM transmission images acquired (**B**) at 280 eV and (**C**) at 500 eV. Identical domain boundaries and positions were marked with the same colour (dashed line) in each image.

In addition, internal structures of those binary colloidal crystals could be observed without destructive sample preparation. Several studies reported the difficulties of structural characterization of binary colloidal crystals due to limitations with respect to the characterization of internal structures or spatial resolution^25,43,44^. Even if smaller particles covered the voids on the top surface with the regular structure, the internal ones cannot be identical to those on the surface. Indeed, distorted internal structures or internal defects were observed, as shown in Fig. 6 (red arrow). The SEM image (Fig. 6A) of the top layer of colloidal crystals shows that 100 nm SiO_2_ particles filled the interstices between 500 nm PS particles. Based on the observation of the arrangement of PS, the structure or orientation of the colloidal crystal could be inferred. However, the irregular structures of SiO_2_ particles marked as red arrow in the STXM image measured at 280 eV at the pre-edge of the C K-edge in Fig. 6B could not be identified in Fig. 6A because the SiO_2_ particles were beneath the PS particles. In addition, a transition from HCP (0001) to FCC (100) plane orientation was observed in the area marked by a green dashed circle. Figure 6B shows that the colloidal structure consisting of SiO_2_ differs in the first row (i.e., ‘Y’ shape) and second row (i.e., ‘ + ’ shape) of the green dashed circles. However, as stated above, this discrepancy was not identified in the SEM image (Fig. 6A) because the transition originated in the bottom layer of the colloidal crystals and internal structures could not be observed by SEM. As a result, SiO_2_ and PS particles were separately observed and internal structures and defects were directly identified by chemical STXM imaging at different incident photon energy.

**Figure 7:**
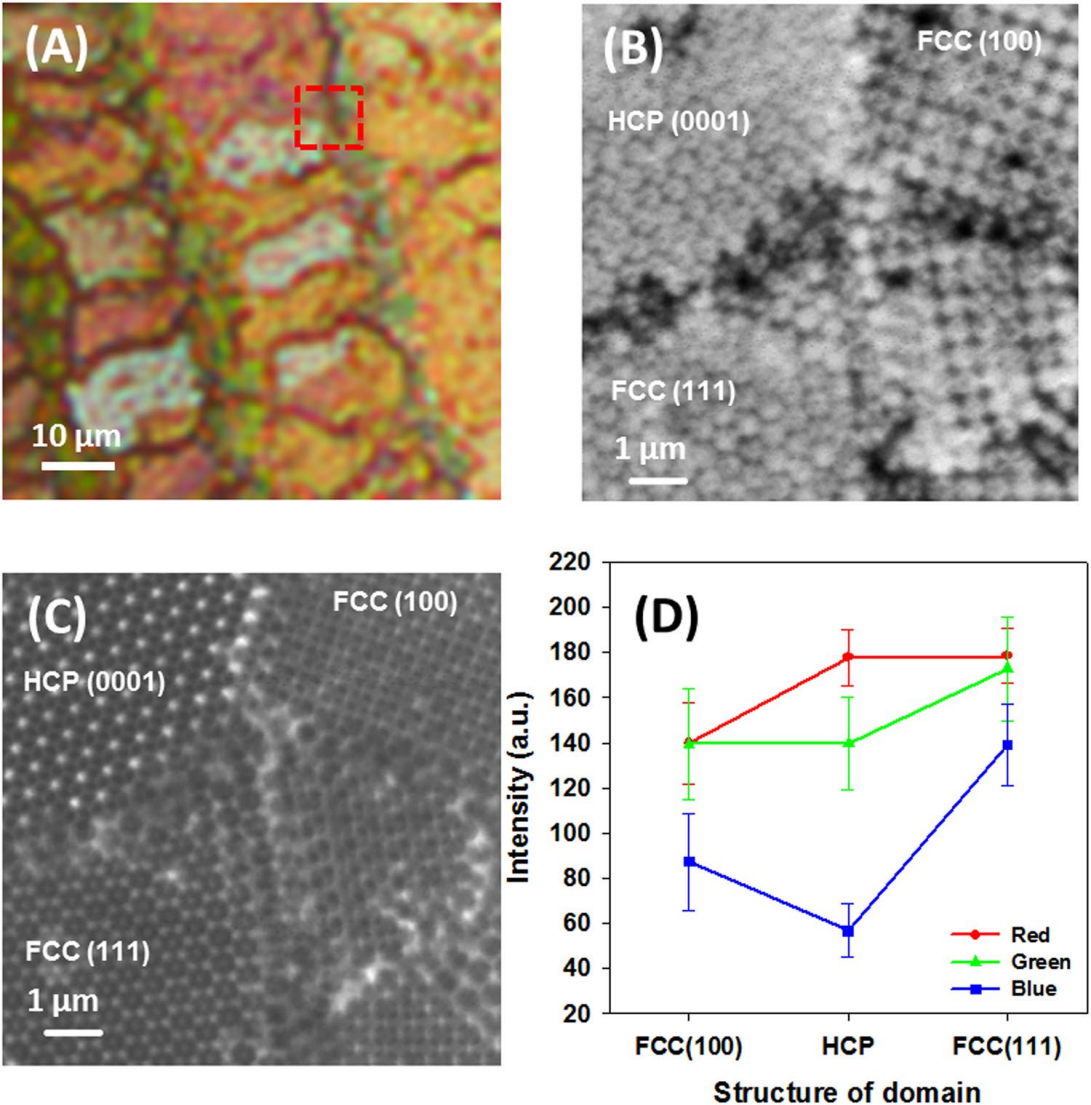
Images of binary colloidal crystals of 100 nm SiO_2_ and 500 nm PS observed by (**A**) OM, (**B**) STXM at 280 eV, and (**C**) at 500 eV. (**D**) Red, green, and blue colour intensity in each domain of FCC (100), HCP (0001), and FCC (111).

The optical properties of binary colloidal crystals in different domains are characterized by colourful OM images, similar to unary crystals. The OM images and collected RGB intensities are shown in Fig. 7A and D. Additionally, the colloidal structures in the region marked with a red dashed box in Fig. 7A could be recognized in Fig. 7B and C. There are three types of domains with structures similar to the structures represented in Fig. 5, that is, FCC (100), HCP (0001), and FCC (111). Therefore, we could compare the colours and structures of binary colloidal crystals. The colours in each domain are similar to that of unary colloidal crystals. However, a slight increase in the green colour intensity was measured (Fig. 7D). As stated above, the stop band can be calculated with the modified Bragg equation and the absorption wavelength is proportional to n_eff_, which is determined by the filling fraction of the colloids. If small colloids fill the voids of large colloids, n_eff_ should increase and a red shift of the absorption maximum (stop band) is expected. Because the OM image was measured in transmission mode, the red shift of the stop band corresponds with the increasing green colour intensity.

Recently, a STXM-based novel imaging technique, ptychography, was developed for chemical composition imaging with better spatial resolution. Because diffraction patterns are reconstructed to the real space image by phase retrieval algorithms in ptychography, limitations of the spatial resolution originating from X-ray optics can be overcome. As a result, 5 nm structures were observed using this technique^45^. For further research, we consider ptychography with sub-10 nm spatial resolution to acquire images of resolved single colloids of binary colloidal crystals.

## Conclusions

We demonstrated the correlations between structures and colours of local domains of colloidal crystals using STXM and OM. Based on the capabilities of STXM, such as significant penetration depth and chemical speciation capability, the internal structures and defects were directly observed for unary and binary colloidal crystals consisting of PS and SiO_2_. Each component of binary colloidal crystals was measured at the pre-edge of the C K-and O K-edge to separately observe SiO_2_ and PS. The STXM and OM images of each domain of FCC (100), FCC (111), and HCP (0001) having different colours in unary colloidal crystals were compared to determine the correlations of structures, crystal orientations, and colours. Due to the local discrepancy of orientations and void volumes of colloidal crystals, the different FCC (100), FCC (111), and HCP (0001) show different colours. Observations using STXM are used complementary to OM and SEM to directly confirm the internal structures of colloidal crystals with simple measurements and minimized preparation. Therefore, we suggest that STXM can be used as the breakthrough tool to characterize colloidal crystals consisting of multiple components.

## Electronic supplementary material

SUPPLEMENTARY INFO
