# Supplementary material for: Structural colour of unary and binary colloidal crystals probed by scanning transmission X-ray microscopy and optical microscopy
Source: Sci Rep. 2017 Sep 29;7:12424. doi: 10.1038/s41598-017-12831-4 (PMC5622058; doi:10.1038/s41598-017-12831-4)
Supplement: Supplementary file 1 — SUPPLEMENTARY INFO [file 41598_2017_12831_MOESM1_ESM.pdf]

Supplementary Information

Structural colour of unary and binary colloidal crystals probed by scanning transmission X-ray microscopy and optical microscopy

Hyun Woo Nho[1,#] *and Tae Hyun Yoon*[1,*]

[1]Department of Chemistry, College of Natural Sciences, Research Institute for Natural Sciences, Hanyang University, Seoul 04762, Republic of Korea

[#] Current Address: LG Chem R&D Campus Daejeon, Daejeon 34122, Republic of Korea

*Corresponding author: Tae Hyun Yoon

Nanoscale Characterization & Environmental Chemistry Lab,

Department of Chemistry, College of Natural Sciences

Hanyang University, Seoul, Korea

E-mail: thyoon@gmail.com

Phone: 82-2-2220-4593

Fax: 82-2-2299-0762

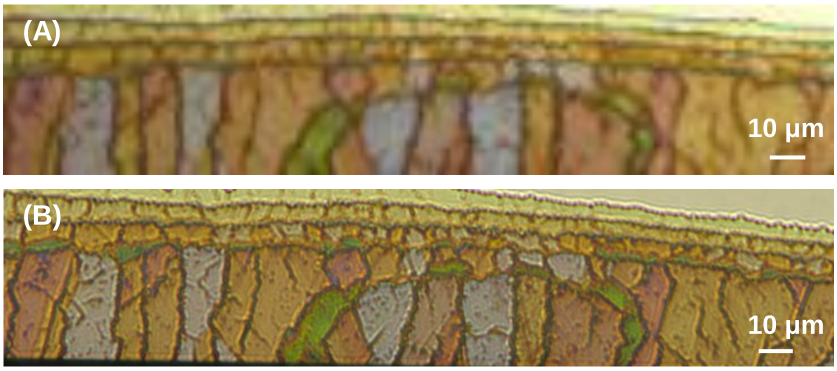

ESI-Figure 1. OM images acquired with (A) 10x objective (NA = 0.3) and (B) 50x objective (NA = 0.8). There are no significant differences in observed colours, although there are differences in spatial resolution.

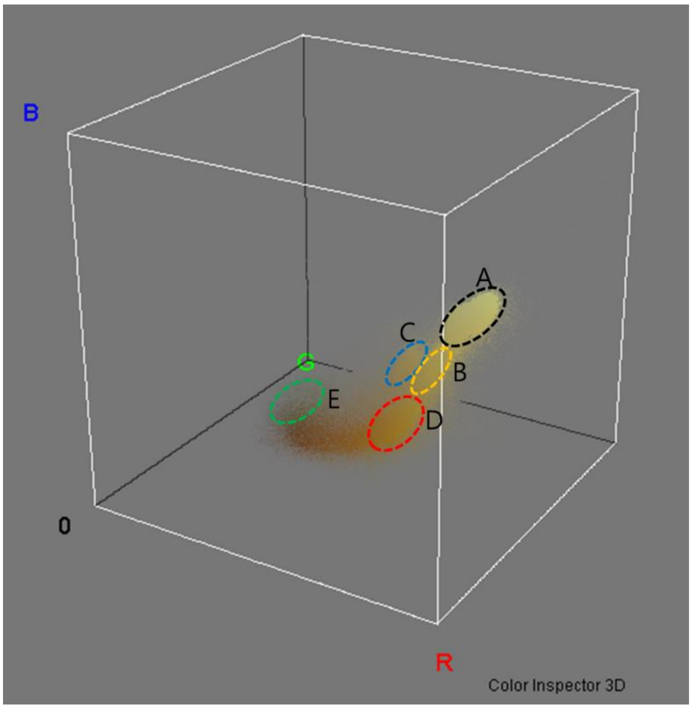

ESI-Figure 2. RGB intensity of each pixel of Figure 3A. (A) Single layer, (B) Double layer, (C) FCC (111), (D) HCP (0001), and (E) FCC (100) domain of colloidal crystals showing different colour intensities. The 3D graph plotted with Colour inspector 3D (plug-in software of ImageJ) in frequency-weighted mode.

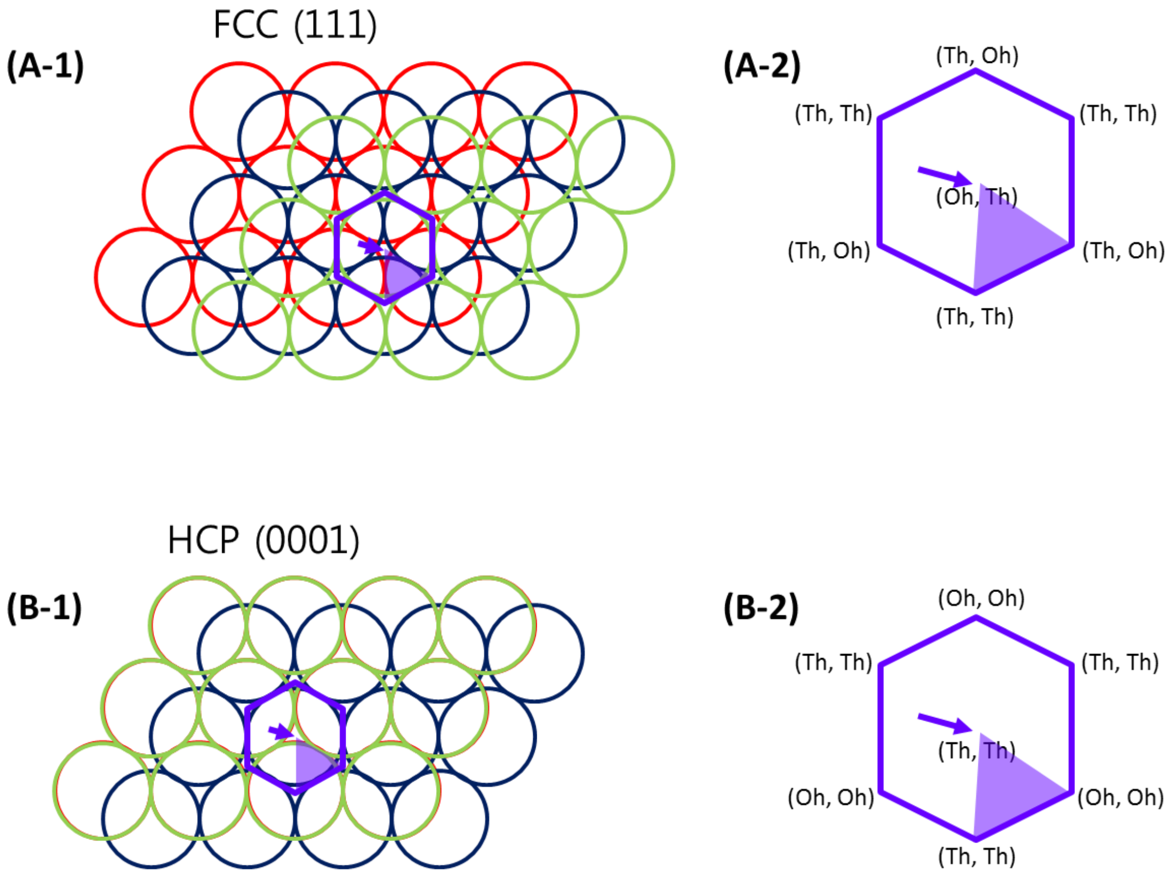

ESI-Figure 3. Analysis of the void sites in the FCC (111) and HCP (0001) structures of colloidal crystals. Model structures of (A-1) FCC (111) and (B-1) HCP (0001). The first, second, and third layers of colloids represented as red, blue, and green circles, respectively. Combination of void types (voids between the first and the second layer, voids in the second and third layer) in the region marked with a purple line in the (A-2) FCC (111) and (B-2) HCP (0001) structure.

ESI-Table 1. Theoretical and measured distance of voids in different structures of colloidal crystals.

		FCC(100)	HCP(0001)	FCC(111)
a	Theoretical distance (nm)	500	866.0	500
	Experimental measurement (nm)	487.4	835.5	477.9
b	Theoretical distance (nm)	353.6	500	288.7
	Experimental measurement (nm)	344.2	477.2	283.0
c	Theoretical distance (nm)	500	500	288.7
	Experimental measurement (nm)	459.9	473.5	271.8
